# Investigation into Cervical Spine Biomechanics Following Single, Multilevel and Hybrid Disc Replacement Surgery with Dynamic Cervical Implant and Fusion: A Finite Element Study

**DOI:** 10.3390/bioengineering9010016

**Published:** 2022-01-04

**Authors:** Muzammil Mumtaz, Iman Zafarparandeh, Deniz Ufuk Erbulut

**Affiliations:** 1 Engineering Center for Orthopaedic Research Excellence (ECORE), Departments of Bioengineering and Orthopaedic Surgery, Colleges of Engineering and Medicine, The University of Toledo, Toledo, OH 43606, USA; muzammilmumtaz24@gmail.com; 2 Department of Biomedical Engineering, Medipol University, Istanbul 34810, Turkey; izafarparandeh@gmail.com; 3 Herston Biofabrication Institute, Metro North Hospital and Health Service, Brisbane, QLD 4029, Australia

**Keywords:** cervical spine, finite element, dynamic cervical implant, multilevel fusion, hybrid surgery, disc replacement

## Abstract

Cervical fusion has been a standard procedure for treating abnormalities associated with the cervical spine. However, the reliability of anterior cervical discectomy and fusion (ACDF) has become arguable due to its adverse effects on the biomechanics of adjacent segments. One of the drawbacks associated with ACDF is adjacent segment degeneration (ASD), which has served as the base for the development of dynamic stabilization systems (DSS) and total disc replacement (TDR) devices for cervical spine. However, the hybrid surgical technique has also gained popularity recently, but its effect on the biomechanics of cervical spine is not well researched. Thus, the objective of this FE study was to draw a comparison among single-level, bi-level, and hybrid surgery with dynamic cervical implants (DCIs) with traditional fusion. Reductions in the range of motion (ROM) for all the implanted models were observed for all the motions except extension, compared to for the intact model. The maximum increase in the ROM of 42% was observed at segments C5–C6 in the hybrid DCI model. The maximum increase in the adjacent segment’s ROM of 8.7% was observed in the multilevel fusion model. The maximum von Mises stress in the implant was highest for the multilevel DCI model. Our study also showed that the shape of the DCI permitted flexion/extension relatively more compared to lateral bending and axial rotation.

## 1. Introduction

Cervical disc degenerative diseases are often treated with traditional anterior cervical discectomy and fusion (ACDF). However, ACDF leads to the loss of motion at the index segment which is compensated by adjacent segments [1]. Thus, the traditional cervical fusion procedure has been under question in recent decades, as studies have reported high rates of adjacent segment degeneration (ASD) following fusion surgery [2]. Hence, motion-preserving devices are being explored by clinicians that may restore motion at the index segment and reduce the chances of developing ASD [2,3,4,5,6]. Dynamic stabilization systems (DSS) and total disc replacement (TDR) implants are commonly used as motion-preserving devices [7,8,9]. TDR devices are mainly composed of ball and socket joint configurations with a constrained or unconstrained center of rotation. The latest TDR devices are single-piece such as simplify. However, TDR implants have been usually associated with the hypermobility at the index segment and causing strain to the surrounding ligaments [10,11]. Thus, dynamic stabilization systems such as dynamic cervical implants (DCIs) serve as intermediate devices between ACDF and TDR. DCIs tend to provide some mobility at the index segment due to its “U-shape” that permits motion in the flexion/extension mostly. Besides hypermobility, TDR implants often yield wear debris due to the sliding contact between articulating surfaces [12]. DCIs are introduced to prevent wear debris, as there are no articulating surfaces.

Single-level disc replacement has become one of the most common procedures for treating abnormalities associated with the intervertebral disc, and its effect on cervical spine biomechanics has been reported in the literature [1,11,13]. On the other hand, bi-level dynamic stabilization systems have also gained attention in the past decades, but very few devices are approved by the FDA for multilevel usage. The DCI is one of the FDA-approved implants for multilevel usage. However, DCIs are less researched compared to other TDR implants. In addition, DCIs are relatively less mobile compared to ball-socket TDR designs. The effect of DCIs with single-level disc replacement is presented in our conference proceedings [14]. In this study, the scope of our work has been extended to evaluate the biomechanics of single-levwl, multilevel and hybrid disc replacement surgery with DCIs and cervical fusion.

**Figure 1:**
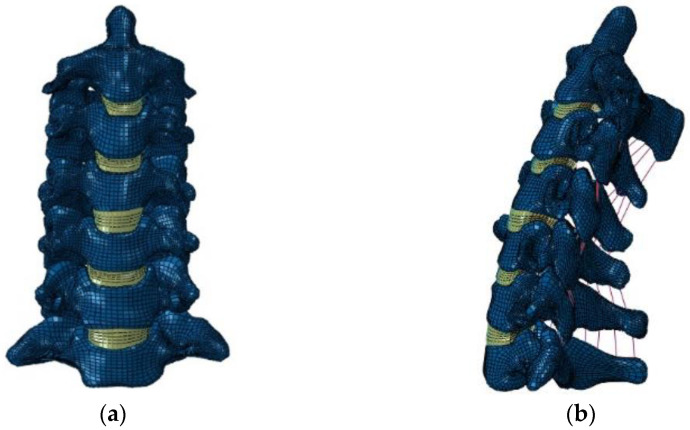
Finite element (FE) model of intact cervical spine segments C2–C7: (**a**) coronal view; (**b**) sagittal view.

**Figure 2:**
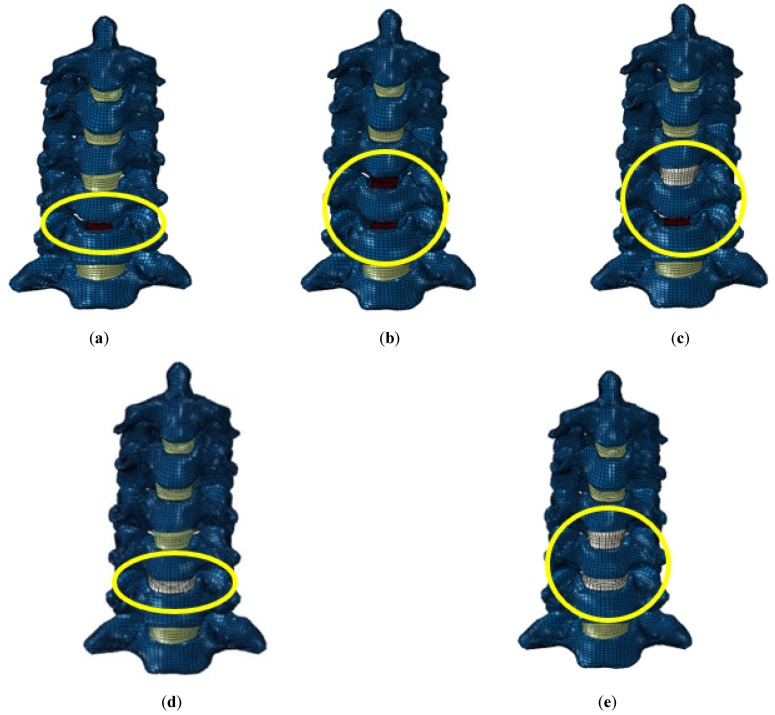
(**a**) C5–C6 dynamic cervical implant (DCI) model; (**b**) C4–C6 DCI model; (**c**) hybrid surgery model; (**d**) C5–C6-fused model; (**e**) C4–C6-fused model.

**Figure 3:**
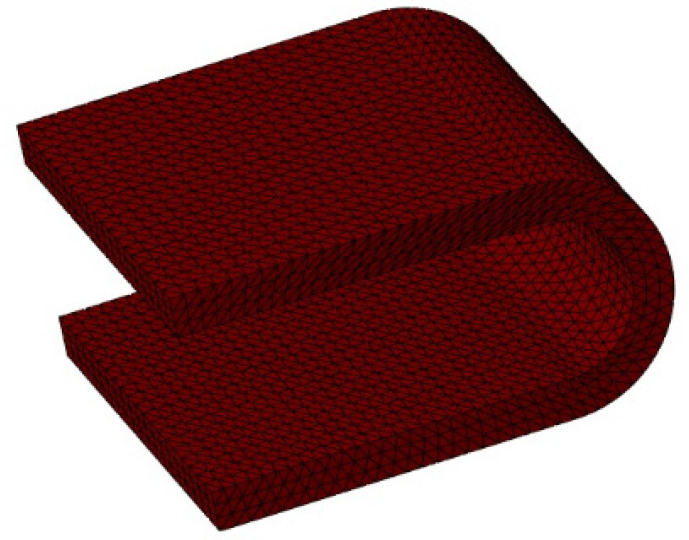
Dynamic cervical implant.

Thus, the aim of our present study was to use finite element (FE) analysis to evaluate the effect of the DCI on the adjacent segment’s range of motion (ROM). For that purpose, five different configurations of the cervical FE model were developed: (1) single-level disc replacement (C5–C6 DCI); (2) bi-level disc replacement (C4–C6 DCI); (3) hybrid surgery (hybrid DCI), C4–C5 fusion, and C5–C6 DCIs; (4) single-level fusion (C5–C6-fused); (5) bi-level fusion (C4–C6-fused), (Figure 1, Figure 2 and Figure 3). The ROMs in all models were calculated and compared with that in the intact model. Moreover, the maximum von Mises stresses in the implant were also measured and compared for all the cases.

## 2. Materials and Methods

A previously validated C2-T1 FE model was used in this study [14,15,16]. In summary, the model was developed based on the computed tomography (CT) scan data of a 35-year-old male. Ethical approval was given by the Institutional Ethics Board committee of the author’s institute. The CT scan data were processed in medical image processing software (Mimics^®^ Version 14.1; Materialise, Inc., Leuven, Belgium) to obtain the three-dimensional (3D) geometry of the vertebrae. The space between the 3D geometry of vertebrae was filled to represent the intervertebral discs. The 3D geometries of the vertebrae and intervertebral discs were meshed with hexahedral elements in IA-FE Mesh software (University of Iowa, IA, USA). The meshed vertebrae and intervertebral discs were exported to ABAQUS software (ABAQUS^®^, Version 6.10-2; Abaqus, Inc., Providence, RI, USA) for FE analysis. To model the behavior of ligaments, 3D truss elements in Abaqus software were used to simulate the nonlinear behavior of ligaments under tensile forces [17,18]. The 3D tension-only truss elements were used to model the five cervical ligaments, i.e., the anterior longitudinal ligament (ALL), the posterior longitudinal ligament (PLL), the capsular ligament (CL), the ligamentum flavum (LF), and the interspinous ligament (ISL). The facets joints were modeled via 3D gap contact elements (GAPUNI) in the Abaqus software.

### 2.1. FE Model of the Instrumented Cervical Spine

To simulate the DCI surgery, the intervertebral disc at the index segment was completely removed along with the ALL and PLL ligaments. A DCI was then inserted into the cavity at the index segment. The upper and lower surfaces of the implant were attached to the respective vertebra via the “TIE” constraint in ABAQUS. The DCI was assigned the material property of a Titanium Alloy (Ti6Al4V) with a Poisson’s ratio of 0.35 and an elastic modulus of 114,000 MPa. The material properties for all the soft and hard tissues in the FE model are summarized in Table 1.

To simulate a single-level disc replacement, the disc between segments C5 and C6 was removed. Similarly, a bi-level disc replacement was simulated by removing the discs and ligaments between segments C4–C6. To simulate hybrid surgery (HS), a disc between segments C5 and C6 was replaced by an implant, segments C4 and C5 were fused by removing the ALL and PLL, and the disc between segments C4 and C5 was assigned the material property of cancellous bone. Similarly, single-level and bi-level fusion was simulated by fusing segments C5–C6 and segments C4–C6, respectively, with assigned cancellous bone properties. 

### 2.2. Loads and Boundary Conditions

A pure moment of 2 Nm was applied at C2 in three planes, i.e., sagittal, coronal, and axial, to simulate flexion and extension, lateral bending (LB), and axial rotation (AR). The T1 bottom surface was fixed under all loading conditions. 

### 2.3. Data Analysis

The postprocessing of the data obtained from FE simulations was performed in MATLAB (MathWorks, Natick, MA, USA) via custom scripts. The ROM for each segment was calculated and compared with the ROM of the intact cervical spine. The percentage difference between the intact and implanted models was calculated with the following equation:

(1) $$Percentage\;Difference=\frac{Implanted_{Data}-Intact_{Data}}{Intact_{Data}}\times 100.$$

## 3. Results

### 3.1. ROM

**Figure 4:**
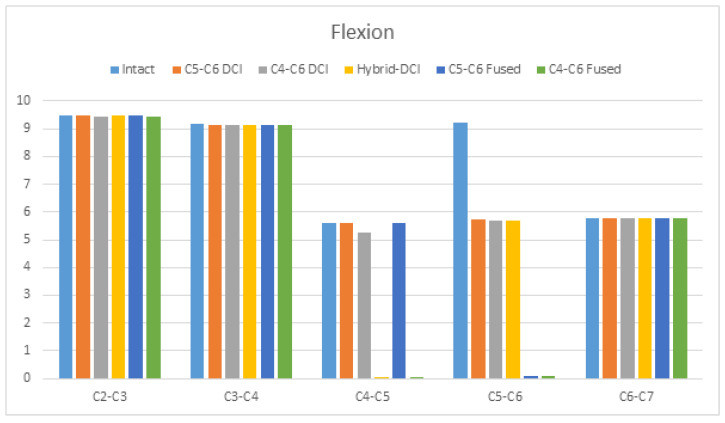
The comparison of the ranges of motion (ROMs) during flexion among the C5–C6 DCI, C4–C6 DCI, hybrid DCI, C5–C6-fused, and C4–C6 fused models. The vertical axis shows ROMs in degrees.

Under flexion, the ROM was reduced by about 38% at segments C5–C6 in C5–C6 DCI, C4–C6 DCI, and hybrid DCI models compared to in the intact model. The ROM was reduced by about 5.9% at segments C4–C5 in the C4–C6 DCI model compared to in the intact model. The changes in the adjacent segment’s ROM were less than 1% for all the surgery models compared to for the intact model (Figure 4).

**Figure 5:**
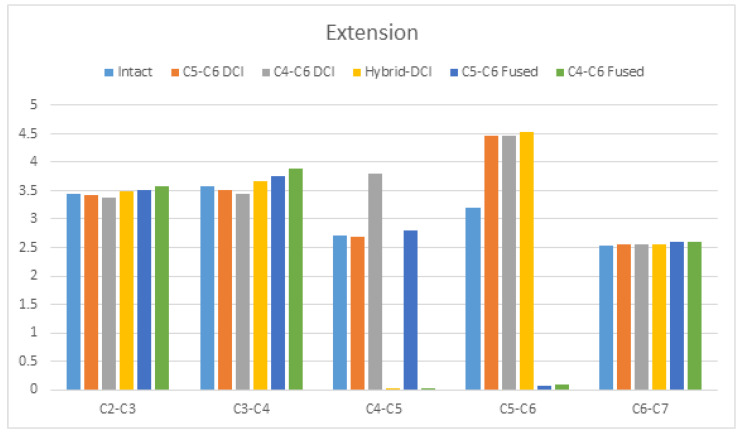
The comparison of ROMs during extension among the C5–C6 DCI, C4–C6 DCI, hybrid DCI, C5–C6-fused, and C4–C6 fused models. The vertical axis shows ROMs in degrees.

Under extension, the maximum increase in ROM of 42% was observed at the C5–C6 DCI in the hybrid DCI model, and that of about 39.7% was observed in the C5–C6 DCI and C4–C6 DCI models. The maximum increase in the adjacent segment’s ROM of about 8.71% was observed at the superior segment in the C4–C6-fused model. The hybrid DCI model had a 2.56% increase in the ROM at the superior segment compared to in the intact model. Conversely, reductions of 1.35% and 3.87% were observed at the superior adjacent segment in the C5–C6 DCI and C4–C6 DCI models, respectively, compared to in the intact model (Figure 5). 

**Figure 6:**
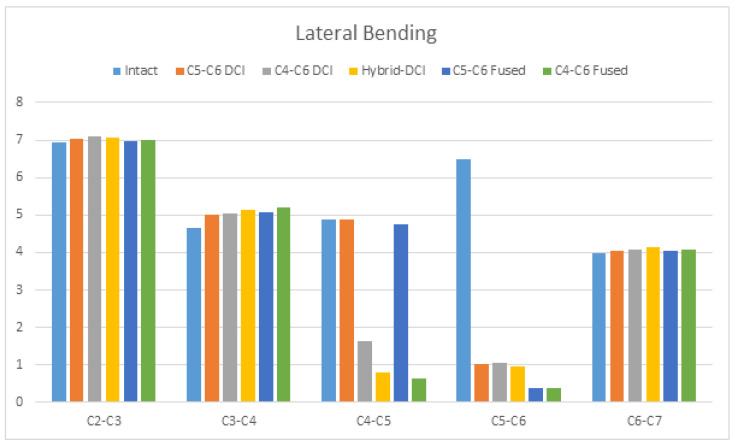
The comparison of ROMs during lateral bending among the C5–C6 DCI, C4–C6 DCI, hybrid DCI, C5–C6-fused, and C4–C6-fused models. The vertical axis shows ROMs in degrees.

Under LB, the C4–C6 DCI model had 66.6% and 83.70% reductions in the ROM at the C4–C5 and C5–C6 segments, respectively, compared to the intact model. The ROM reductions of about 84.25% and 85.39% were observed at segments C5–C6 in the C5–C6 DCI and hybrid DCI models, respectively, compared to in the intact model. The maximum increase in the adjacent segment’s ROM was observed at the superior adjacent segment of the C4–C6-fused model, i.e., 11.35%, compared to in the intact model. Moreover, a 10.28% increase in ROM at the superior adjacent segment in the hybrid DCI model was observed, compared to in the intact model (Figure 6). 

**Figure 7:**
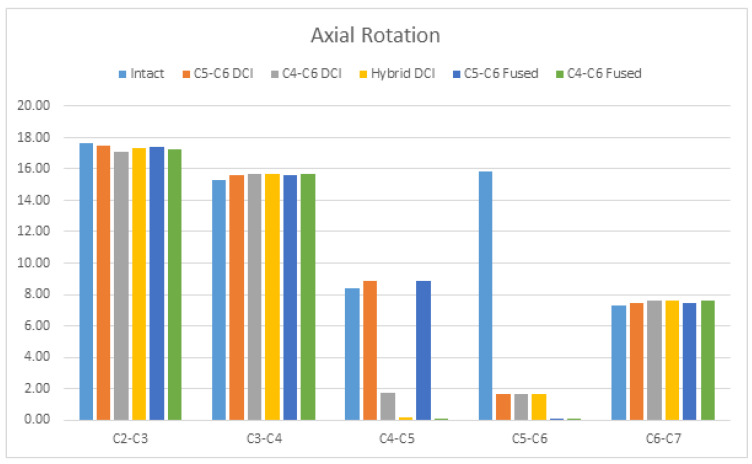
The comparison of ROMs during axial rotation among the C5–C6 DCI, C4–C6 DCI, hybrid DCI, C5–C6-fused, and C4–C6-fused models. The vertical axis shows ROMs in degrees.

Under AR, an about 89% reduction in ROM was observed in all the implanted models at segments C5–C6, whereas segments C4–C5 in the C4–C6 DCI model had a 79.42% reduction, compared to in the intact model. Conversely, an increase in the ROM was observed at the adjacent segments of all the models, compared to in the intact model. The superior adjacent segment in the C5–C6 DCI and C5–C6-fused models showed increases in the ROM of 5.92% and 6.34%, respectively, compared to in the intact model. The adjacent segment’s ROMs for other implanted models were increased by less than 4%, compared to for the intact model (Figure 7).

### 3.2. Stress Distribution in the Implant

**Figure 8:**
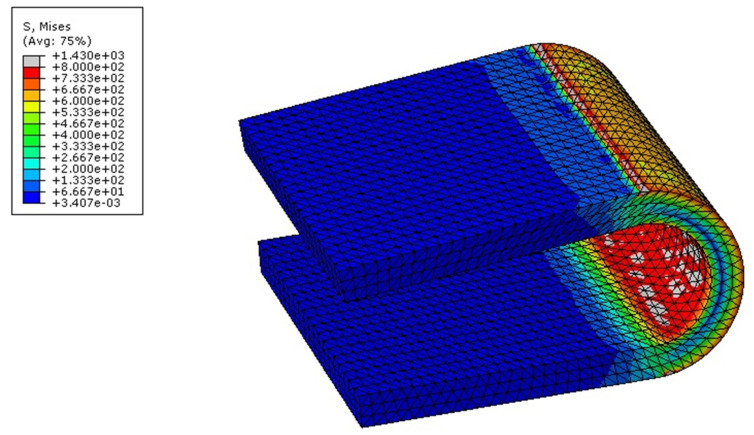
Maximum stress distribution in the U-shaped implant (DCI) during flexion in the C4–C6 DCI model.

The maximum von Mises stresses predicted in the implant for different models are summarized in Figure 8. The maximum von Mises stress was observed during flexion in all the models, and it was highest in the C4–C6 DCI model, i.e., 765.85 MPa at segments C5–C6. During extension, the maximum stress of 633.64 MPa was predicted in the hybrid DCI model. Under LB, the maximum stress of 575.62 MPa was predicted during the right lateral bending (RLB) at segments C4–C5 of the C4–C6 DCI model. Under AR, the maximum stress observed was about 648.5 MPa at segments C5–C6 and C4–C6 of the C5–C6 DCI and C4–C6 DCI models, respectively.

## 4. Discussion

DSS and TDR implants have gained popularity in recent decades, as they tend to reduce the radiographic ASD by preserving motion at the index segment. These motion-preserving implants employ different mechanisms for preserving the motion at the operated segment. However, some of TDR devices tend to increase ROMs more than natural ROMs at the index segment, as reported by Chang et al. and Kotani et al. [11,19]. Besides this, some studies have also reported that the use of TDR may lead to excessive loading at the facet joints [20,21]. For such reasons, DCIs have drawn significant attention of medical professionals as an alternate to traditional TDR devices. However, the literature on biomechanics of cervical spine following DCI surgery is sparse per the author’s knowledge. Hence, the aim of this FE analysis was to study the effect of DCIs on cervical spine biomechanics and compare it with the effect of the traditional fusion surgery. 

ACDF

The fusion models demonstrated reductions of above 90% motion for all the directions and a maximum reduction of up to 99% motion at the index segment were observed. The maximum reductions in the ROM for single-level and bi-level fusion models were observed during AR. The adjacent segment’s ROM generally increased more for the bi-level fusion model as compared to for the single-level fusion model. Prasarn et al. conducted an in vitro investigation of single-level vs. multilevel ACDF on 10 fresh frozen cadavers and concluded that the bi-level ACDF increases more motion at adjacent segments compared to the single-level ACDF [22]. The increase in the adjacent segment’s ROM may accelerate the ASD and may require surgical intervention. 

Thus, Veeravagu et al. conducted a clinical administrative database study and found that revision surgeries are more common in subjects that undergo multilevel ACDF as compared to subjects who undergo single-level ACDF surgery [23]. The results of our FE study also demonstrated higher chances of the adjacent segment breakdown in the bi-level fusion model.

DCI

The results of our FE analysis showed that the DCIs in all the models caused reductions of the ROM at the index segment during flexion and increased the ROM during extension, which is a similar trend to that observed by Li et al. [24]. The increase in the extension ROM and the decrease in the flexion ROM could be attributed to the shape of the DCI. As the U-shape of the DCI had a cavity that opened to the anterior side of the cervical spine, this can cause the implant to pivot about the posterior wall of the U-shaped DCI during extension. Conversely, during flexion, the posterior wall of the U-shaped DCI resisted the motion, leading to higher stresses in flexion (Figure 8). Therefore, the maximum von Mises stress was also predicted during flexion in all the implanted models, and our finding was also in agreement with the study by Mo et al. [25]. The highest maximum von Mises stress predicted in the DCI implant was predicted for the C4–C6 DCI model, and it was higher than the endurance limit of Ti6Al4V (500 MPa), suggesting implant failure may occur relatively earlier than other DCI configurations. 

Besides this, the DCI had less effect on the adjacent segment’s ROM during flexion/extension and more effect during LB and AR. Mo et al. simulated a single-level DCI at the C5–C6 level and observed a similar trend in their computational analysis [26]. Li et al. simulated a bi-level DCI model and observed that the DCI has a very less effect on the adjacent segment’s ROM during flexion/extension [24]. However, they did not simulate LB and AR, which prevents the authors from making a comparison with other motions.

Hybrid Surgery

The hybrid surgery model showed a higher motion at the index segment (C5–C6) during extension compared to the single-level and bi-level DCI models. The adjacent segment’s ROM for the hybrid surgery model was slightly higher than the DCI models and lower than the fusion models. The hybrid surgery yielded ROM results that were intermediary between ACDF and TDR. This finding of our study is consistent with the computational analysis of Wong et al. [25]

### 4.1. Limitations

This FE study had limitations due to the nature of the mathematical modeling technique. Material properties, interactions, boundary conditions, and loads very used in the model were simplified. The effect of the neck musculature was also not simulated. These limitations may alter the biomechanics of cervical spine and the stress on the implants. However, the general trends for the ROM and the implant stress may remain the same.

### 4.2. Conclusions

In summary, this study used computational analysis to draw a comparison among single-level DCI, bi-level DCI, and hybrid DCI configurations with single- and bi-level fusion. Our biomechanical FE study suggested that the DCI may be a good alternate to ACDF, but it had its own limitations. The DCI preserved the ROM reasonably for flexion/extension, but the stress in the implant was also maximum during flexion. The ROM of the adjacent segment was least affected in the C5–C6 DCI model. Besides this, the DCI tended to experience a high stress in the bi-level (C4–C6 DCI) model, suggesting that failure may occur sooner in patients with multilevel DCI implantation compared to in patients with single-level or hybrid DCI implantation.

## Figures and Tables

**Table 1:** Material properties for different components of the cervical spine finite element (FE) model [15].

Component				
Bony Structure	Element Type	Young’s Modulus (MPa)	Poisson’s Ratio	Cross-Sectional Area (mm^2^)
Vertebral cortical bone	Isotropic, elastic hex element	10,000	0.30	-
Vertebral cancellous bone	Isotropic, elastic hex element	450	0.25	-
Ligaments				
Transverse, tectorial membrane	Isotropic, elastic hex element	80	0.3	-
Apical–alar	Tension-only, truss elements	20	0.3	-
Anterior longitudinal	Tension-only, truss elements	15 (<12%) 30 (>12%)	0.3	11.1
Posterior longitudinal	Tension-only, truss elements	10 (<12%) 20 (>12%)	0.3	11.3
Ligamentum flavum	Tension-only, truss elements	5 (<25%) 10 (>25%)	0.3	46.0
Capsular	Tension-only, truss elements	7 (<30%) 30 (>12%)	0.3	42.2
Joint				
Facet (apophyseal joint)	Nonlinear soft contact, GAPUNI	-	-	-

## Data Availability

The data presented in this study are available on request from the corresponding author.
